# Supplementary material for: MetaRibo-Seq measures translation in microbiomes
Source: Nat Commun. 2020 Jun 29;11:3268. doi: 10.1038/s41467-020-17081-z (PMC7324362; doi:10.1038/s41467-020-17081-z)
Supplement: Supplementary file 10 — Supplementary Data 7 [file 41467_2020_17081_MOESM10_ESM.zip › File2/Confidence_High_Taxonomy/435870_out.krona.html]

Javascript must be enabled to view this page.

members
magnitude
magnitudeUnassigned
count
unassigned
taxon
rank

435870\_out

312

superkingdom
312
2

phylum
312
976

class
312
200643

171549
order
312

815
family
312

816
312

SRS013638\_contig\_number\_9997SRS014683\_contig\_number\_16550SRS015217\_contig\_number\_24583SRS015369\_contig\_number\_4250SRS015486\_contig\_number\_437SRS020328\_contig\_number\_22216SRS022137\_contig\_number\_13493SRS049995\_contig\_number\_22384SRS050941\_contig\_number\_12332SRS051882\_contig\_number\_10147SRS053335\_contig\_number\_18098SRS063370\_contig\_number\_8855SRS074670\_contig\_number\_4262SRS078176\_contig\_number\_15362SRS098073\_contig\_number\_15421SRS100021\_contig\_number\_8946SRS1041091\_contig\_number\_22583SRS1041095\_contig\_number\_2907SRS1041138\_contig\_number\_13662SRS1041147\_contig\_number\_1666SRS146764\_contig\_number\_4775SRS148511\_contig\_number\_12420SRS893300\_contig\_number\_9742SRS893369\_contig\_number\_209SRS971275\_contig\_number\_36670
25
genus

18
species

SRS019601\_contig\_number\_12726SRS049959\_contig\_number\_17082SRS050422\_contig\_number\_35146SRS055966\_contig\_number\_1891SRS065176\_contig\_number\_11208SRS074586\_contig\_number\_830SRS077086\_contig\_number\_564SRS077127\_contig\_number\_9705SRS077194\_contig\_number\_12974SRS101433\_contig\_number\_10787SRS1041137\_contig\_number\_25430SRS1041140\_contig\_number\_19783SRS144135\_contig\_number\_33845SRS146813\_contig\_number\_15611SRS147377\_contig\_number\_8970SRS893292\_contig\_number\_6960SRS893295\_contig\_number\_6082SRS893366\_contig\_number\_9020
2292941

species
1
1638779

SRS893358\_contig\_number\_4720

162156

SRS045244\_contig\_number\_335
species
1

2292944

SRS015065\_contig\_number\_17772SRS018427\_contig\_number\_contig-100\_643.177678SRS042966\_contig\_number\_16415SRS050026\_contig\_number\_1444SRS1055056\_contig\_number\_9421SRS142980\_contig\_number\_6718
species
6

2302926

SRS143991\_contig\_number\_contig-100\_893.176209
species
1

10
species

SRS016897\_contig\_number\_201SRS019381\_contig\_number\_1839SRS021153\_contig\_number\_480SRS024075\_contig\_number\_212SRS046068\_contig\_number\_337SRS052027\_contig\_number\_13687SRS054905\_contig\_number\_4703SRS063489\_contig\_number\_1101SRS893253\_contig\_number\_206SRS893256\_contig\_number\_1602
818

10
species

SRS013687\_contig\_number\_26598SRS023971\_contig\_number\_310SRS045739\_contig\_number\_212SRS049900\_contig\_number\_300SRS075773\_contig\_number\_21038SRS076876\_contig\_number\_163SRS1055069\_contig\_number\_484SRS142503\_contig\_number\_28178SRS144506\_contig\_number\_34859SRS893382\_contig\_number\_166
46506


SRS065397\_contig\_number\_2170
310297
1
species

3
species

SRS045826\_contig\_number\_21003SRS075878\_contig\_number\_8180SRS143523\_contig\_number\_23046
671267

4
species

SRS077730\_contig\_number\_399SRS098644\_contig\_number\_11402SRS098881\_contig\_number\_5135SRS142599\_contig\_number\_13746
817

821

SRS011084\_contig\_number\_5335SRS011239\_contig\_number\_5482SRS011302\_contig\_number\_8092SRS011405\_contig\_number\_15572SRS012902\_contig\_number\_163SRS012969\_contig\_number\_22996SRS013158\_contig\_number\_287SRS013382\_contig\_number\_638SRS013521\_contig\_number\_3078SRS013965\_contig\_number\_27281SRS014287\_contig\_number\_11010SRS014313\_contig\_number\_2009SRS014412\_contig\_number\_6100SRS014459\_contig\_number\_10544SRS014534\_contig\_number\_276SRS014736\_contig\_number\_685SRS014855\_contig\_number\_4959SRS014979\_contig\_number\_27338SRS015190\_contig\_number\_15888SRS015431\_contig\_number\_51581SRS015663\_contig\_number\_7987SRS015694\_contig\_number\_18146SRS015782\_contig\_number\_15985SRS015854\_contig\_number\_9527SRS016056\_contig\_number\_262SRS016132\_contig\_number\_175SRS016495\_contig\_number\_3224SRS016629\_contig\_number\_1616SRS016753\_contig\_number\_1385SRS016840\_contig\_number\_967SRS016954\_contig\_number\_4482SRS016989\_contig\_number\_138SRS017103\_contig\_number\_2109SRS017191\_contig\_number\_6006SRS017247\_contig\_number\_1934SRS017307\_contig\_number\_14219SRS017375\_contig\_number\_135SRS017433\_contig\_number\_14165SRS017745\_contig\_number\_453SRS017859\_contig\_number\_253SRS018623\_contig\_number\_11902SRS018623\_contig\_number\_12063SRS018817\_contig\_number\_13500SRS018836\_contig\_number\_24673SRS018888\_contig\_number\_4241SRS019030\_contig\_number\_11255SRS019068\_contig\_number\_3821SRS019178\_contig\_number\_359SRS019267\_contig\_number\_292SRS019397\_contig\_number\_2407SRS019445\_contig\_number\_16629SRS019496\_contig\_number\_10302SRS019693\_contig\_number\_2283SRS019808\_contig\_number\_12264SRS019968\_contig\_number\_contig-100\_625.104619SRS020394\_contig\_number\_14451SRS020508\_contig\_number\_49SRS021219\_contig\_number\_2192SRS021484\_contig\_number\_6540SRS022524\_contig\_number\_15830SRS022713\_contig\_number\_248SRS023829\_contig\_number\_164SRS023914\_contig\_number\_7706SRS024009\_contig\_number\_7316SRS024492\_contig\_number\_4437SRS024625\_contig\_number\_10657SRS043411\_contig\_number\_2274SRS043768\_contig\_number\_30906SRS044535\_contig\_number\_2990SRS045645\_contig\_number\_304SRS046369\_contig\_number\_8159SRS046502\_contig\_number\_13691SRS046810\_contig\_number\_contig-100\_91.92SRS048164\_contig\_number\_13400SRS049402\_contig\_number\_8752SRS049712\_contig\_number\_8864SRS049773\_contig\_number\_15005SRS050520\_contig\_number\_212SRS050752\_contig\_number\_6822SRS050801\_contig\_number\_3771SRS050974\_contig\_number\_contig-100\_93.24132SRS050998\_contig\_number\_12005SRS051031\_contig\_number\_30788SRS051610\_contig\_number\_1681SRS052576\_contig\_number\_22SRS053087\_contig\_number\_contig-100\_222.44668SRS053214\_contig\_number\_7080SRS053356\_contig\_number\_46496SRS053649\_contig\_number\_18490SRS054059\_contig\_number\_4421SRS054352\_contig\_number\_15852SRS055017\_contig\_number\_10900SRS055982\_contig\_number\_3607SRS056537\_contig\_number\_10154SRS057478\_contig\_number\_1859SRS057480\_contig\_number\_10355SRS058268\_contig\_number\_61SRS058723\_contig\_number\_1023SRS062427\_contig\_number\_4149SRS062654\_contig\_number\_9000SRS062701\_contig\_number\_4894SRS063127\_contig\_number\_458SRS063190\_contig\_number\_9263SRS063813\_contig\_number\_4863SRS063928\_contig\_number\_102SRS064557\_contig\_number\_354SRS064645\_contig\_number\_541SRS064757\_contig\_number\_8286SRS064973\_contig\_number\_7SRS075078\_contig\_number\_19511SRS075398\_contig\_number\_363SRS075716\_contig\_number\_4172SRS075821\_contig\_number\_2612SRS075984\_contig\_number\_11712SRS076756\_contig\_number\_347SRS076976\_contig\_number\_4900SRS077294\_contig\_number\_14734SRS077335\_contig\_number\_1764SRS077454\_contig\_number\_7742SRS077552\_contig\_number\_1680SRS077589\_contig\_number\_1320SRS077641\_contig\_number\_4919SRS077991\_contig\_number\_contig-100\_56.23963SRS078665\_contig\_number\_2305SRS097889\_contig\_number\_33984SRS097958\_contig\_number\_1767SRS098061\_contig\_number\_3293SRS098571\_contig\_number\_13096SRS098827\_contig\_number\_1767SRS100907\_contig\_number\_contig-100\_64.62506SRS104036\_contig\_number\_472SRS104084\_contig\_number\_6890SRS1041031\_contig\_number\_8766SRS1041032\_contig\_number\_14790SRS1041036\_contig\_number\_651SRS1041037\_contig\_number\_16971SRS1041090\_contig\_number\_2044SRS1041092\_contig\_number\_2005SRS1041116\_contig\_number\_4243SRS1041118\_contig\_number\_4489SRS1041129\_contig\_number\_4169SRS1041130\_contig\_number\_866SRS1041133\_contig\_number\_24493SRS1041134\_contig\_number\_10011SRS1041139\_contig\_number\_2255SRS1041141\_contig\_number\_126SRS1041142\_contig\_number\_7288SRS1041143\_contig\_number\_70SRS1041157\_contig\_number\_5339SRS104327\_contig\_number\_5229SRS104400\_contig\_number\_1283SRS104485\_contig\_number\_14SRS104693\_contig\_number\_8389SRS104912\_contig\_number\_11274SRS104975\_contig\_number\_1347SRS105082\_contig\_number\_9396SRS105153\_contig\_number\_15572SRS1054716\_contig\_number\_11716SRS1055022\_contig\_number\_12413SRS1055038\_contig\_number\_807SRS1055049\_contig\_number\_4205SRS1055076\_contig\_number\_1386SRS1055083\_contig\_number\_118SRS1055095\_contig\_number\_832SRS140513\_contig\_number\_10095SRS142542\_contig\_number\_4071SRS142781\_contig\_number\_1972SRS142890\_contig\_number\_22493SRS142923\_contig\_number\_5047SRS143070\_contig\_number\_2856SRS143085\_contig\_number\_5058SRS143148\_contig\_number\_24098SRS143342\_contig\_number\_2162SRS143372\_contig\_number\_1520SRS143417\_contig\_number\_1199SRS143722\_contig\_number\_10502SRS143876\_contig\_number\_13343SRS143895\_contig\_number\_29983SRS143991\_contig\_number\_19430SRS144183\_contig\_number\_22063SRS144297\_contig\_number\_8018SRS144362\_contig\_number\_20381SRS144537\_contig\_number\_21080SRS144714\_contig\_number\_1857SRS144753\_contig\_number\_contig-100\_33.87394SRS145308\_contig\_number\_315SRS147022\_contig\_number\_1952SRS147039\_contig\_number\_508SRS147271\_contig\_number\_4897SRS147425\_contig\_number\_5888SRS147557\_contig\_number\_33277SRS147614\_contig\_number\_contig-100\_358.68569SRS147766\_contig\_number\_1513SRS147977\_contig\_number\_560SRS148091\_contig\_number\_11416SRS148196\_contig\_number\_34705SRS148253\_contig\_number\_6668SRS148319\_contig\_number\_13096SRS148784\_contig\_number\_16239SRS148817\_contig\_number\_2308SRS148970\_contig\_number\_371SRS149075\_contig\_number\_10245SRS149244\_contig\_number\_383SRS892882\_contig\_number\_12326SRS893172\_contig\_number\_2103SRS893187\_contig\_number\_17980SRS893259\_contig\_number\_1887SRS893279\_contig\_number\_2669SRS893285\_contig\_number\_161SRS893288\_contig\_number\_213SRS893327\_contig\_number\_356SRS893341\_contig\_number\_10977SRS893373\_contig\_number\_18860SRS893378\_contig\_number\_19049SRS893380\_contig\_number\_275
species
215


SRS016095\_contig\_number\_7845SRS017521\_contig\_number\_16494SRS077849\_contig\_number\_6336SRS140492\_contig\_number\_12209
291645
4
species


SRS047044\_contig\_number\_9401
310298
1
species

1
species

SRS049446\_contig\_number\_4292
2292939

4
species

SRS020622\_contig\_number\_3527SRS097920\_contig\_number\_11034SRS1041164\_contig\_number\_384SRS1054928\_contig\_number\_1951
246787

338188

SRS042284\_contig\_number\_1812SRS893170\_contig\_number\_734
species
2


SRS016203\_contig\_number\_3012SRS019910\_contig\_number\_25953SRS074964\_contig\_number\_853SRS077502\_contig\_number\_6212
47678
4
species

626930

SRS149325\_contig\_number\_14003
species
1
